# Morphological Changes of the Suboccipital Musculature in Women with Myofascial Temporomandibular Pain: A Case-Control Study

**DOI:** 10.3390/life13051159

**Published:** 2023-05-11

**Authors:** Daniel Ulman-Macón, César Fernández-de-las-Peñas, Santiago Angulo-Díaz-Parreño, José L. Arias-Buría, Juan A. Mesa-Jiménez

**Affiliations:** 1 Department of Physical Therapy, Universidad San-Pablo CEU, 28660 Madrid, Spain; danielulman@hotmail.com (D.U.-M.); sangulo@ceu.es (S.A.-D.-P.); jmesaj@ceu.es (J.A.M.-J.); 2 Department of Physical Therapy, Occupational Therapy, Rehabilitation and Physical Medicine, Universidad Rey Juan Carlos, Alcorcón, 28922 Madrid, Spain; cesar.fernandez@urjc.es; 3 Cátedra Institucional en Docencia, Clínica e Investigación en Fisioterapia, Terapia Manual, Punción Seca y Ejercicio Terapéutico, Universidad Rey Juan Carlos, Alcorcón, 28922 Madrid, Spain; 4 Máster Oficial en Dolor Orofacial y Disfunción Cráneo-Mandibular, Universidad San-Pablo CEU, 28660 Madrid, Spain

**Keywords:** temporomandibular pain, ultrasound, suboccipital muscles, cross sectional area

## Abstract

Temporomandibular disorder (TMD) is an umbrella term including pain problems involving the cranio-cervical region. It has been suggested that patients with TMD also exhibit cervical spine disturbances. Evidence suggests the presence of morphological changes in the deep cervical muscles in individuals with headaches. The objective of this study was to compare the morphology of the suboccipital muscles between women with TMD and healthy controls. An observational, cross-sectional case-control study was conducted. An ultrasound examination of the suboccipital musculature (rectus capitis posterior minor, rectus capitis posterior major, oblique capitis superior, oblique capitis inferior) was conducted in 20 women with myofascial TMD and 20 matched controls. The cross-sectional area (CSA), perimeter, depth, width, and length of each muscle were calculated by a blinded assessor. The results revealed that women with myofascial TMD pain exhibited bilaterally reduced thickness, CSA, and perimeter in all the suboccipital muscles when compared with healthy women. The width and depth of the suboccipital musculature were similar between women with myofascial TMD and pain-free controls. This study found morphological changes in the suboccipital muscles in women with myofascial TMD pain. These changes can be related to muscle atrophy and are similar to those previously found in women with headaches. Future studies are required to investigate the clinical relevance of these findings by determining if the specific treatment of these muscles could help clinically patients with myofascial TMD.

## 1. Introduction

Temporomandibular disorders (TMDs) include craniofacial pain conditions involving the temporomandibular joint, masticatory muscles, and associated neuromusculoskeletal structures of the head and neck [1]. TMD represents the most important cause of non-dental pain in the orofacial region. Patients with TMD usually present pain, limited jaw movement, and joint sounds [1,2]. Although this term represents a group of both painful and painless musculoskeletal disorders, painful TMD is the most common form of chronic orofacial pain in the general population, affecting up to 5% to 12% [3]. Painful TMD can be divided into myofascial TMD and arthralgic TMD, depending on the origin of the symptoms. Manfredini et al. found that the prevalence of TMD from myofascial origin was 45.3%, while the prevalence of TMD from the joint origin, i.e., disc displacement, was up to 41.1% [4]. Poveda-Roda et al. also found that myofascial TMD was the most common diagnosis (42%) [5]. Additionally, a Spanish study reported that the prevalence of myofascial TMD pain was 46.7%, of which 25.2% reported symptoms during the last year [6].

The association between the cervical spine and TMD pain has been discussed for decades. A recent systematic review concluded that there is a clinical association between TMD and the cervical spine [7]. This meta-analysis found a limited active range of motion of the cervical spine (moderate evidence), reduced muscle endurance of the cervical flexors (limited evidence), and motor control impairments manifested as changes in recruitment of the neck and orofacial musculature (conflicting evidence) in individuals with TMD, particularly those of myofascial origin [7]. The association between TMD pain and the cervical spine is anatomically explained by the relationship between the trigeminal nerve and the upper cervical spine nerves into the trigemino-cervical nucleus caudalis [8]. In the trigemino-cervical nucleus caudalis, nociceptive afferents from the upper cervical spinal nerves (C1–C3 segments) converge onto second-order neurons that also receive afferents from the trigeminal nerve (particularly the first division, V1), via the trigeminal spinal tract which permits interchange of inputs between the upper cervical segments and the first (ophthalmic) branch of the trigeminal nerve [8]. This anatomical relationship would explain the fact that any tissue, e.g., joint, muscle, ligament, cranial dura, innervated by the trigemino-cervical nucleus caudalis, can cause pain in the head and face [8].

In the last decades, imaging technology has contributed to explaining the seemingly disconnected spectrum of biopsychosocial signs and symptoms of some musculoskeletal pain conditions of the cervical spine [9]. Several studies using Magnetic Resonance Imaging (MRI) have identified the presence of morphological changes in the cervical extensors, particularly the deep muscles, such as the multifidus, in people with neck and head pain symptoms. For instance, patients with whiplash exhibit fatty infiltration of the rectus capitis minor and major muscles and multifidi muscles [10]. These studies demonstrated that these morphological changes occur soon after the injury and are associated with poor long-term recovery suggesting their role in the chronification of pain [9,10]. However, the presence of morphological changes in the cervical musculature in headache populations is heterogeneous. Fernández-de-las-Peñas et al. observed a reduction of the cross-sectional area of the rectus capitis posterior minor and major muscles in patients with chronic tension-type headaches when compared with healthy controls [11]. Additionally, these morphological changes were associated with the frequency and intensity of the headache attacks supporting an association between pain and muscle atrophy [11]. Hvedstrup et al. did not find significant differences in the cross-sectional area of the rectus capitis posterior minor muscle between patients with migraine and healthy controls [12]. On the contrary, Yuan et al. identified hypertrophy, i.e., increases in cross-sectional area, of the rectus capitis posterior minor muscle in a sample of chronic headache patients without specifying which type of headache the patients suffered from [13]. It is possible that morphological changes in the cervical muscles are present in chronic pain conditions with a higher musculoskeletal pain component, e.g., tension-type headaches.

Most studies examining neck musculature morphology had used MRI, an equipment not readily available in clinical practice for daily use. Ultrasound imaging has gained relevance in recent years in the evaluation of cervical spine musculature. In fact, different studies reported the ability of ultrasound imaging to investigate deep neck extensors, such as the cervical multifidus [14] and suboccipital [15] muscles. No previous study has investigated cervical muscle morphology in individuals with TMD. Accordingly, the objective of the current study was to compare muscle morphology, as assessed with ultrasound imaging, of the suboccipital musculature: rectus capitis posterior minor (RCPmin), rectus capitis posterior major (RCPmaj), oblique capitis superior (OCS) and oblique capitis inferior (OCI), between a sample of women with myofascial TMD and healthy controls. We hypothesized that women with myofascial TMD exhibit morphological changes in the suboccipital musculature, particularly a decreased cross-sectional area when compared to healthy pain-free controls.

## 2. Methods

### 2.1. Study Design

This observational, cross-sectional case-control study was conducted in accordance with the Declaration of Helsinki and was approved by the Human Research Local Ethics Committee of Universidad Rey Juan Carlos University (Spain) (URJC 02219412.5.0000). All participants provided their informed consent before collecting data.

### 2.2. Participants

Consecutive patients with TMD pain symptoms presenting to a private physiotherapy clinic were screened for eligibility criteria. To be eligible to participate, patients should have to be females, aged over 40 years, and with a diagnosis of myofascial TMD according to the Diagnostic Criteria for TMD (DC/TMD) [3]. The following symptomatology was assessed using the DC/TMD criteria: location of pain, jaw range of motion and associated joint pain, clicking sounds, and pain upon muscle and joint palpation. 

In addition, age- and sex-matched healthy pain-free controls were also included. In order to be eligible for this group, subjects should present no history of TMD pain and no history of neck pain the previous year prior to the assessment. The same examination of the orofacial area, including jaw range of motion, clicking sounds, and muscle and joint palpations, was also conducted in the healthy control group. 

The exclusion criteria for both groups included: 1, history of cervical trauma; 2, cervical surgery; 3, cervical radiculopathy; 4, diagnosis of fibromyalgia; 5, pregnancy; 6, previous history of alcohol or drug abuse; 7, had received any physical therapy treatment the last three months.

### 2.3. Ultrasound Assessment

All ultrasound examinations were conducted with a Mindray M7 ultrasound equipment with a 12 MHz linear probe (Shenzhen Mindray Bio-Medical Electronics Co., Ltd., Shenzhen, China) by an examiner with more than 10 years of experience in musculoskeletal imaging and blinded to the subject’s condition as follows.

The participant was placed in the prone position, with the head inside the hole in the stretcher, with the headrest inclined 20° to respect the thoracic kyphosis, and the arms at 90° of shoulder abduction. Ultrasound images of each of the suboccipital muscles were obtained according to previous guidelines.

**Figure 1:**
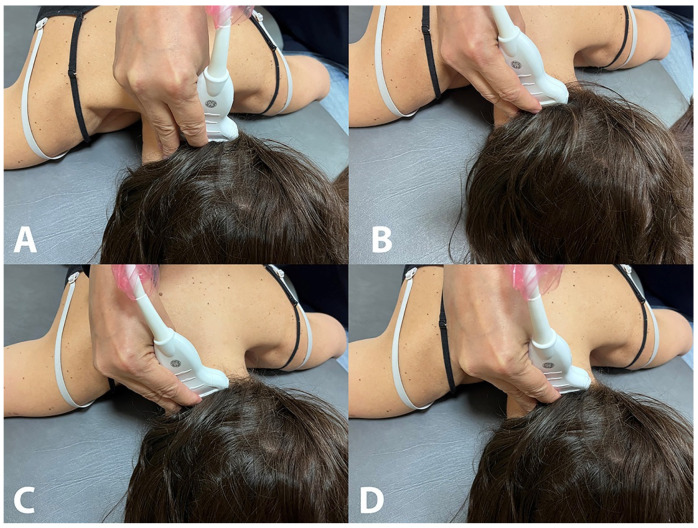
Placement of the ultrasound probe for the assessment of the rectus capitis posterior minor (**A**), rectus capitis posterior major (**B**), oblique capitis superior (**C**) and oblique capitis inferior (**D**).

**Figure 2:**
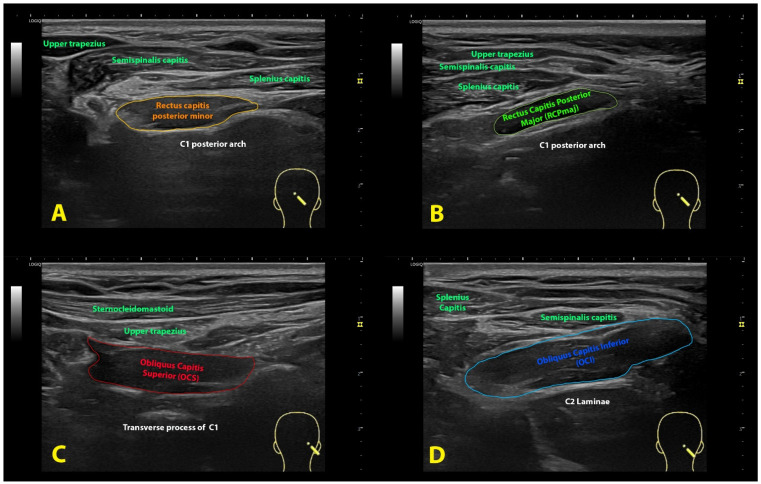
Ultrasound imaging of the rectus capitis posterior minor (**A**), rectus capitis posterior major (**B**), oblique capitis superior (**C**) and oblique capitis inferior (**D**).

Rectus Capitis Posterior Minor (RCPmin): The RCPmin muscle originates at the tubercle on the posterior arch of the atlas and attaches to the medial part of the inferior nuchal line and to the occipital bone between the inferior nuchal line and the foramen magnum. For its examination, the spinous process of C2 was identified by manual palpation. The ultrasound probe was moved laterally until identifying the lamina of C2. In this position, the probe was moved up until the lamina of C1 vertebra was identified and moved slightly medial to identify the posterior arch of C1 (Figure 1A). In this position, the probe was moved up/down to identify the muscular borders of the RCPmin in a transverse plane [16] (Figure 2A).

Rectus Capitis Posterior Major (RCPmaj): The RCPmaj muscle originates at the spinous process of C2 and attaches to the lateral part of the inferior nuchal line and the occipital bone immediately below it. For its examination, the spinous process of C2 was identified by manual palpation. The ultrasound probe was moved laterally until identifying the lamina of C2 (Figure 1B). In this position, the probe was moved up until the lamina of C1 vertebra was identified and moved up/down to identify the borders of the RCPmaj in a transverse plane (Figure 2B) [17]. This procedure has shown moderate inter-rater reliability and excellent intra-rater reliability [18].

Oblique Capitis Superior (OCS): The OCS muscle originates at the upper surface of the transverse process of C1 and attaches to the occipital bone between the superior and inferior nuchal lines lateral to the semispinalis capitis muscle. The procedure was similar to the RCPmaj but more lateral. The spinous process of C2 was identified by palpation. The ultrasound probe was moved laterally until identifying the lamina of C2 and moved up until the lamina of C1 vertebra was identified. In this position, the probe was moved laterally to the transverse process of C1 (Figure 1C) and moved up/down to identify the borders of the OCS in a transverse plane (Figure 2C) [17].

Oblique Capitis Inferior (OCI): The OCI muscle originates adjacent to the upper part of the lamina of C1 and attaches to the inferior-posterior aspect of the transverse process of C2. For its examination, the spinous process of C2 and the transverse process of C1 were identified by manual palpation, and the ultrasound probe was aligned with the short axis of the OCI muscle (Figure 1D) to identify the borders of the muscle (Figure 2D) [19].

### 2.4. Imaging Management

**Figure 3:**
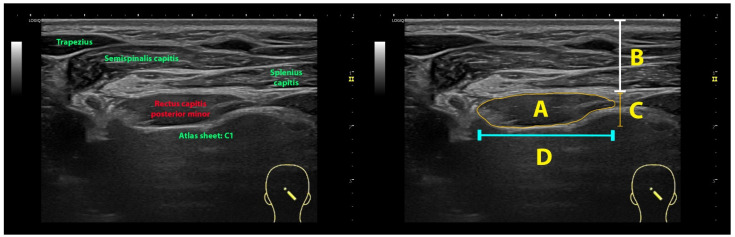
Imaging management: cross-sectional area (A), depth (B), thickness (C) and width (D) using the rectus capitis posterior minor muscle as example.

All images were codified, processed, and analyzed in the offline DICOM ImageJ software v. 1.42 (National Institutes of Health, Bethesda, MD, USA). The cross-sectional area (CSA), perimeter, depth, width, and thickness of each of the suboccipital muscles was assessed by an experienced assessor with more than 15 years in ultrasound imaging and blinded to the subject’s condition (Figure 3).

### 2.5. Statistical Analysis

Data were analyzed with the Statistical Package for the Social Sciences (SPSS) version 27.0 (SPSS Inc., Chicago, IL, USA). Descriptive statistics (mean and standard deviation) are presented. A normal distribution of quantitative data was assessed by means of the Kolmogorov–Smirnov test (*p* > 0.05). An analysis of variance (ANOVA) with side (right, left) as within-subjects variable and group (myofascial TMD, controls) as between-subjects variables were used to determine between-groups differences in the ultrasound parameters. A two-sided *p*-value of less than 0.05 indicated statistical significance.

## 3. Results

Twenty women with myofascial TMD pain (age: 49 ± 6 years; weight: 65 kg ± 11 kg; height: 165 ± 7 cm.) and 20 matched healthy controls (age: 49 ± 7 years: weight: 66 ± 10 kg; height: 166 ± 8 cm.) were included. Women with myofascial TMD pain reported a history of chronic pain for more than six months. In addition, 80% (*n* = 16) of them reported headaches associated with TMD.

### 3.1. Rectus Capitis Posterior Minor (RCPmin) Muscle

The two-way ANOVA revealed a group effect for thickness (F = 13.707, *p* < 0.001), CSA (F = 17.373, *p* < 0.001), and perimeter (F = 13.707, *p* < 0.001), but not for width (F = 0.171, *p* = 0.681) and depth (F = 0.384, *p* = 0.537) of the RCPmin muscle. No significant group * side effect was observed for thickness (F = 0.841, *p* = 0.362), width (F = 0.006, *p* = 0.937), depth (F = 0.368, *p* = 0.546), CSA (F = 0.749, *p* = 0.390) and perimeter (F = 0.841, *p* = 0.362). Women with TMD of myofascial origin exhibited bilaterally reduced thickness, CSA, and perimeter but similar width and depth of the RCPmin muscle to healthy women (Table 1).

### 3.2. Rectus Capitis Posterior Major (RCPmaj) Muscle

A significant group effect for thickness (F = 18.331, *p* < 0.001), CSA (F = 19.718, *p* < 0.001), and perimeter (F = 18.331, *p* < 0.001), but not for width (F = 0.112, *p* = 0.739) and depth (F = 0.562, *p* = 0.456) was found for the RCPmaj muscle. Again, no significant group * side effect was either found: thickness (F = 1.523, *p* = 0.221), width (F = 0.007, *p* = 0.934), depth (F = 0.018, *p* = 0.893), CSA (F = 1.237, *p* = 0.270) and perimeter (F = 1.523, *p* = 0.221). Thickness, CSA, and perimeter of the RCPmaj were bilaterally lower in women with myofascial TMD pain than in healthy women (Table 2).

### 3.3. Oblique Capitis Superior (OCS) Muscle

The two-way ANOVA found a significant group effect for thickness (F = 12.376, *p* < 0.001), CSA (F = 18.357, *p* < 0.001), and perimeter (F = 19.376, *p* < 0.001) but not for width (F = 0.075, *p* = 0.785) and depth (F = 0.508, *p* = 0.478) of the OCS muscle. No significant group * side effect on either parameter of the OCS was identified: thickness (F = 0.403, *p* = 0.527), width (F = 0.001, *p* = 0.974), depth (F = 0.001 *p* = 0.970), CSA (F = 0.286, *p* = 0.595) and perimeter (F = 0.403, *p* = 0.527). The OCS of women with myofascial TMD exhibited bilateral reduced thickness, CSA, and perimeter but similar width and depth to the OCS of healthy women (Table 3).

### 3.4. Oblique Capitis Inferior (OCI) Muscle

The two-way ANOVA also revealed a significant group effect for thickness (F = 9.512, *p* = 0.003), CSA (F = 7.542, *p* = 0.006), and perimeter (F = 9.512, *p* = 0.003), but not for width (F = 0.447, *p* = 0.506) and depth (F = 0.527, *p* = 0.470) of the OCI muscle. No group * side effect for thickness (F = 1.630, *p* = 0.206), width (F = 0.966, *p* = 0.329), depth (F = 0.025, *p* = 0.874), CSA (F = 1.577, *p* = 0.213) and perimeter (F = 1.630, *p* = 0.206) of the OCI was seen (Table 4).

## 4. Discussion

The presence of impairments in the cervical spine in individuals with TMD pain has been previously described [7]. It has been found that patients with TMD, particularly those with myofascial pain, exhibit a reduced range of motion of the cervical spine, positive flexion rotation test, and motor control disturbances expressed as reduced strength and greater fatigability of the cervical muscles [7]. Nevertheless, most studies conducted on individuals with TMD have focused on deep neck flexor performance [20,21]. In fact, all these cervical spine musculoskeletal impairments observed in individuals with TMD are similar to those previously reported in individuals with headaches [7,20,21]. Schomacher and Falla claimed the relevance of the deep neck extensors in musculoskeletal chronic pain conditions of the cervical spine [22]. The current study revealed, for the first time, the presence of morphological changes in the suboccipital musculature in a sample of women with myofascial TMD pain. Overall, women with myofascial TMD pain exhibited reduced thickness, CSA, and perimeter bilaterally in all the suboccipital muscles when compared with healthy women. The width and depth of the suboccipital muscles were similar between women with myofascial TMD and healthy pain-free controls.

Our study provides evidence about the presence of impairments in the cervical spine in individuals with myofascial TMD supporting the presence of morphological changes in the suboccipital musculature. Current findings are similar to that previously observed in individuals with chronic tension-type headaches [11] and with cervicogenic headaches [23]. These studies revealed decreased CSA, in agreement with our results, in the suboccipital muscles, as a potential manifestation of muscle atrophy. However, these studies [11,23] did not evaluate other morphological measures of the suboccipital muscles, not being able to confirm if the observed differences in CSA were due to differences in muscle size or form. The presence of muscular atrophy in our study would be further supported by the fact that the width and depth of the muscles were similar between women with myofascial TMD and healthy pain-free controls, whereas the perimeter, thickness, and CSA were bilaterally reduced. These data suggest that the form of the suboccipital muscles is similar between women with myofascial TMD and pain-free controls and that differences are associated with a change in the size, but not the form, of the musculature (e.g., CSA or thickness). Interestingly, overall data revealed that the suboccipital musculature is symmetrical in both patients with myofascial TMD and healthy people. Accordingly, our results found a pattern of bilateral changes in all the suboccipital muscles within the TMD group. Two previous studies evaluated the RCPmin and OCI in a cohort of healthy individuals and found that these muscles were asymmetrical, with higher values on the right side when compared with the left side [24,25]. Differences in age, sex, lifestyle habits, or study design could explain discrepancies between previous studies [24,25] and the current one. Further, these studies used MRI [24,25], whereas we used US imaging.

The presence of muscle atrophy in the suboccipital musculature has several implications for chronic pain conditions. First, it could act as a potential perpetuating factor for chronic pain. In fact, the suboccipital muscles have a higher concentration of muscle spindles (36 spindles/gram RCPmin; 30.5 spindles/gram RCPmaj) in comparison with superficial cervical muscles such as the splenius capitis (7.6 spindles/gram) [26]. The presence of a high density of muscle spindles suggests that the suboccipital muscles act as proprioceptive monitors of the upper cervical spine. In such a scenario, muscle atrophy could account for a reduction of the proprioceptive output originating from these muscles, hence, facilitating the transmission of impulses from wide dynamic range nociceptors to the spinal cord. The fact that the suboccipital muscles exhibit several attachments to the cranial dura mater throughout a soft tissue connective bridge (called myodural bridge complex) with a high density of nociceptive fibers [27] reinforces their relevance in nociception. However, it seems that this myodural bridge is not present in all individuals [28]. In fact, Sun et al. [29] found significantly smaller CSA of the RCPmin in individuals with a myodural bridge attachment when compared with individuals without this attachment. Second, muscle atrophy may also be the result of a protective mechanism for avoiding further damage of sensible structures (e.g., cranial dura matter), although this seems to be more plausible in other chronic pain conditions more associated with active movements, such as low back pain where atrophy of the lumbar multifidi has also been identified [30]. Third, the morphological changes in the suboccipital musculature could also be a consequence of a forward head posture since this position of the head consists of an extension of the upper cervical spine, which can lead to a shortening of the suboccipital musculature. If this were the case, not only the size but also the form would have been different, which was not the case. Further, the evidence does not support a clear association between TMD and forward head posture [31]. Finally, the presence of morphological changes in the suboccipital muscles could also be related to upper cervical spine impairments. For instance, Greenbaum et al. observed a positive cervical flexion-rotation test in individuals with myofascial TMD but not in patients with arthrogenic TMD [32,33]. A positive cervical flexion-rotation test is associated with a reduced range of motion of the upper cervical spine and potential dysfunction of C1. Since the OCI muscle is the main rotator muscle of the C1–C2 joint, it is possible that upper cervical spine impairment contributes to muscle atrophy by a sensitization process.

The clinical relevance of the suboccipital musculature is supported by the fact that manual interventions targeting the upper cervical spine are effective in decreasing TMD related-pain [32]. A recent meta-analysis showed that manual therapy targeting the cervical spine was effective (moderate evidence) in decreasing TMD pain and increasing pain-free maximal mouth opening in individuals with TMD [34]. The presence of muscle atrophy in the suboccipital would also suggest that patients with myofascial TMD could benefit from therapeutic exercise on the cervical spine, as previously suggested [35]. Accordingly, exercise programs targeting the deep neck extensor muscles could be implemented in this patient population in combination with exercise programs targeting the deep neck flexors [34,35].

Although this is the first study investigating morphological changes in the suboccipital muscles with ultrasound imaging in patients with TMD pain or myofascial origin, potential limitations should be recognized. First, we included a potentially small sample size; however, the fact that significant differences were observed suggests that the inclusion of a larger sample would not alter the direction of the results. Second, this is a cross-sectional study; therefore, we cannot say if muscle atrophy of the suboccipital muscles is a primary or a secondary phenomenon to TMD pain. As it has been previously commented, atrophy of the suboccipital muscles could be a cause or consequence of chronic pain; nevertheless, independently of this bidirectional association, the presence of muscle atrophy will contribute to the maintenance of pain. Third, although the use of ultrasound imaging has risen in the last decades, it should be recognized that a gold standard for assessing muscle morphology is MRI. Future studies comparing the results between ultrasound imaging and MRI would help to confirm or refute current results.

## 5. Conclusions

This cross-sectional case-control study found that women with myofascial TMD pain exhibited reduced thickness, CSA, and perimeter bilaterally in all the suboccipital muscles as compared with healthy women. The width and depth of the suboccipital musculature were similar between women with myofascial TMD and healthy pain-free controls. Current results suggest the presence of muscle atrophy in the suboccipital muscles, which clinical relevance should be clarified in future studies.

## Figures and Tables

**Table 1:** Cross-sectional area, thickness, perimeter, depth and width of the rectus capitis posterior minor (RCPmin) muscle.

	TMD (*n* = 20)		Healthy Controls (*n* = 20)	
Measure	Right Side	Left Side	Right Side	Left Side
CSA *	0.35 (0.1)	0.3 (0.1)	0.7 (0.25)	0.65 (0.2)
Perimeter *	2.0 (0.3)	1.9 (0.25)	2.9 (0.45)	2.85 (0.4)
Depth	1.45 (0.2)	1.5 (0.1)	1.6 (0.2)	1.6 (0.2)
Width	4.1 (0.1)	4.1 (0.15)	4.2 (0.05)	4.1 (0.1)
Thickness *	0.65 (0.1)	0.6 (0.05)	0.9 (0.15)	0.9 (0.1)

CSA: Cross-sectional area; TMD: Temporomandibular pain. * Statistically significant differences between groups (ANOVA).

**Table 2:** Cross-sectional area, thickness, perimeter, depth and width of the rectus capitis posterior major (RCPmaj) muscle.

	TMD (*n* = 20)		Healthy Controls (*n* = 20)	
Measure	Right Side	Left Side	Right Side	Left Side
CSA *	0.3 (0.1)	0.3 (0.05)	0.75 (0.2)	0.7 (0.2)
Perimeter *	2.0 (0.3)	1.9 (0.25)	3.0 (0.4)	2.9 (0.4)
Depth	1.5 (0.15)	1.5 (0.15)	1.65 (0.2)	1.6 (0.2)
Width	4.1 (0.1)	4.05 (0.2)	4.2 (0.1)	4.15 (0.05)
Thickness *	0.65 (0.1)	0.6 (0.1)	0.95 (0.15)	0.9 (0.1)

CSA: Cross-sectional area; TMD: Temporomandibular pain. * Statistically significant differences between groups (ANOVA).

**Table 3:** Cross-sectional area, thickness, perimeter, depth and width of the oblique capitis superior (OCS) muscle.

	TMD (*n* = 20)		Healthy Controls (*n* = 20)	
Measure	Right Side	Left Side	Right Side	Left Side
CSA *	0.5 (0.3)	0.45 (0.1)	0.85 (0.3)	0.8 (0.25)
Perimeter *	2.2 (0.7)	2.25 (0.3)	2.8 (0.6)	2.9 (0.5)
Depth	1.6 (0.2)	1.5 (0.15)	1.55 (0.2)	1.6 (0.15)
Width	4.15 (0.05)	4.1 (0.1)	4.2 (0.25)	4.15 (0.1)
Thickness *	0.8 (0.2)	0.75 (0.1)	1.0 (0.15)	1.0 (0.2)

CSA: Cross-sectional area; TMD: Temporomandibular pain. * Statistically significant differences between groups (ANOVA).

**Table 4:** Cross-sectional area, thickness, perimeter, depth and width of the oblique capitis inferior (OCI) muscle.

	TMD (*n* = 20)		Healthy Controls (*n* = 20)	
Measure	Right Side	Left Side	Right Side	Left Side
CSA *	0.45 (0.2)	0.45 (0.15)	0.9 (0.25)	0.9 (0.3)
Perimeter *	2.25 (0.5)	2.3 (0.45)	3.3 (0.5)	3.39 (0.5)
Depth	1.5 (0.2)	1.45 (0.15)	1.6 (0.2)	1.55 (0.2)
Width	4.1 (0.15)	4.1 (0.15)	4.2 (0.1)	4.15 (0.05)
Thickness *	0.7 (0.15)	0.75 (0.15)	1.05 (0.15)	1.05 (0.15)

CSA: Cross-sectional area; TMD: Temporomandibular pain. * Statistically significant differences between groups (ANOVA).

## Data Availability

The data presented in this study are available upon reasonable request from the corresponding author.
