# Correction: Ulman-Macón et al. Morphological Changes of the Suboccipital Musculature in Women with Myofascial Temporomandibular Pain: A Case-Control Study. *Life* 2023, *13,* 1159

**DOI:** 10.3390/life14111387

**Published:** 2024-10-28

**Authors:** Daniel Ulman-Macón, César Fernández-de-las-Peñas, Santiago Angulo-Díaz-Parreño, José L. Arias-Buría, Juan A. Mesa-Jiménez

**Affiliations:** 1 Department of Physical Therapy, Universidad San-Pablo CEU, 28660 Madrid, Spain; danielulman@hotmail.com (D.U.-M.); sangulo@ceu.es (S.A.-D.-P.); jmesaj@ceu.es (J.A.M.-J.); 2 Department of Physical Therapy, Occupational Therapy, Rehabilitation and Physical Medicine, Universidad Rey Juan Carlos, Alcorcón, 28922 Madrid, Spain; cesar.fernandez@urjc.es; 3 Cátedra Institucional en Docencia, Clínica e Investigación en Fisioterapia, Terapia Manual, Punción Seca y Ejercicio Terapéutico, Universidad Rey Juan Carlos, Alcorcón, 28922 Madrid, Spain; 4 Máster Oficial en Dolor Orofacial y Disfunción Cráneo-Mandibular, Universidad San-Pablo CEU, 28660 Madrid, Spain

**Error in Figures**

In the original publication [1], there was a mistake in Figure 1 as published. There was an error in the order of the figures and in the figure of the oblique capitis inferior muscle. The corrected Figure 1 appears below.

In the original publication, there was a mistake in Figure 2 as published. There was an error in the labelling of the musculature in the ultrasound description. The corrected Figure 2 appears below.

In the original publication, there was a mistake in Figure 3 as published. There was an error in the labelling of the musculature in the ultrasound description. The corrected Figure 3 appears below.

**Text Correction**

There was an error in the original publication in the anatomical description of the oblique capitis inferior muscle. The original version stated the following:

Oblique Capitis Inferior (OCI): The OCI muscle originates adjacent to the upper part of the lamina of C1 and attaches to the inferior-posterior aspect of the transverse process of C2.

A correction has been made to Section 2.3 Ultrasound Assessment, in the description of the OCI, as follows: 

Oblique Capitis Inferior (OCI): The OCI muscle originates at the spinous process of C2 vertebra and inserts into the posterior aspect of the transverse process of C1.

The authors state that the scientific conclusions are unaffected. This correction was approved by the Academic Editor. The original publication has also been updated.

## Figures and Tables

**Figure 1:**
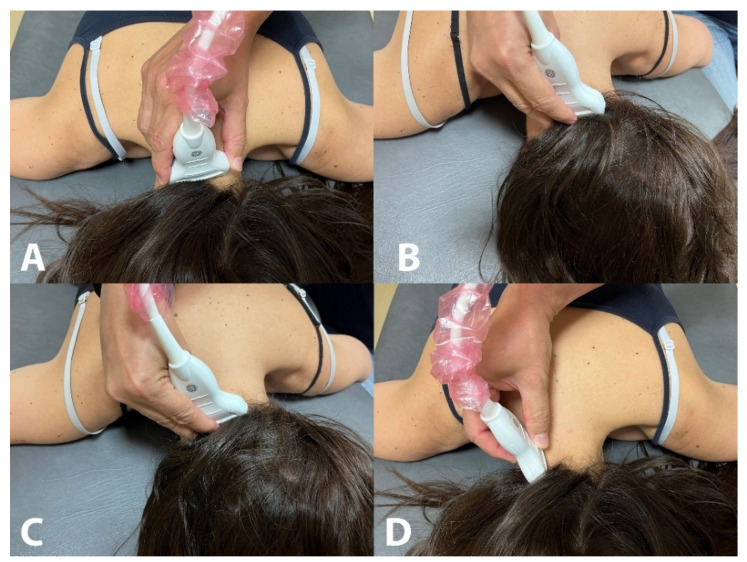
Placement of the ultrasound probe for the assessment of the rectus capitis posterior minor (**A**), rectus capitis posterior major (**B**), oblique capitis superior (**C**) and oblique capitis inferior (**D**).

**Figure 2:**
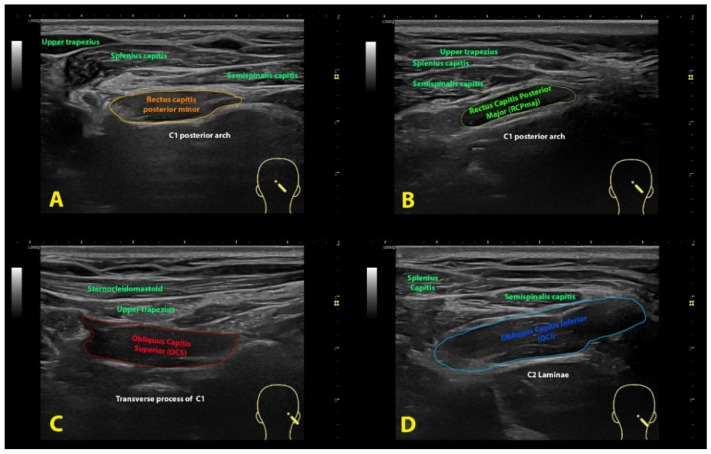
Ultrasound imaging of the rectus capitis posterior minor (**A**), rectus capitis posterior major (**B**), oblique capitis superior (**C**) and oblique capitis inferior (**D**).

**Figure 3:**
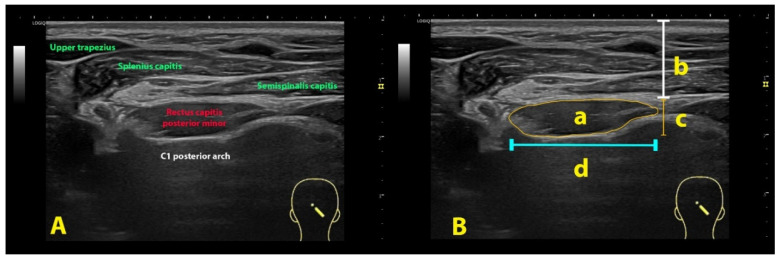
Ultrasound imaging of the rectus capitis posterior minor (**A**) and its imaging management (**B**): cross-sectional area (a), depth (b), thickness (c) and width (d) using the rectus capitis posterior minor muscle as example.
